# Pediatric androgenetic alopecia: a retrospective review of clinical characteristics, hormonal assays and metabolic syndrome risk factors in 23 patients^☆^

**DOI:** 10.1016/j.abd.2021.06.006

**Published:** 2022-01-13

**Authors:** Deren Özcan

**Affiliations:** Department of Dermatology, Başkent University Faculty of Medicine, Ankara, Turkey

**Keywords:** Adolescents, Androgenetic alopecia, Children, Metabolic syndrome

## Abstract

**Background:**

Androgenetic alopecia in the pediatric population is rarely discussed in the literature. Although the prevalence of the metabolic syndrome is increased in patients with early-onset androgenetic alopecia, the presence of metabolic syndrome risk factors in pediatric androgenetic alopecia is unknown.

**Objective:**

To evaluate the demographics, medical and family histories, clinical and trichoscopic features, androgenic hormones, and metabolic syndrome risk factors in pediatric androgenetic alopecia.

**Methods:**

The medical reports of pediatric patients with androgenetic alopecia were reviewed.

**Results:**

The study included 23 patients (12 females and 11 males) with a mean age of 15,3 ± 2,1 years. Sixteen patients had adolescent androgenetic alopecia and seven, had childhood alopecia. Nine patients reported a family history, all of whom had adolescent androgenetic alopecia. Hyperandrogenism was noted in three patients with adolescent androgenetic alopecia. The most common hair loss pattern was diffuse thinning at the crown with preservation of the frontal hairline which was noted in 10 patients (43.5%), six of whom were males. Fourteen patients (60.9%) had at least one metabolic syndrome risk factor. The most common risk factor was obesity or overweight (47.8%) followed by insulin resistance (21.7%), high fasting blood glucose (13%), high blood pressure (4.4%) and lipid abnormalities (4.4%).

**Study limitations:**

Retrospective study; lack of a control group.

**Conclusion:**

Pediatric androgenetic alopecia is often associated with metabolic syndrome risk factors. Therefore, androgenetic alopecia in the pediatric population may indicate a future metabolic syndrome which warrants an accurate and prompt diagnosis for early screening and treatment.

## Introductıon

In children and adolescents, alopecia areata and trichotillomania are reportedly the most common forms of alopecia, but Androgenetic Alopecia (AGA) is suggested to be more frequent than was previously anticipated.^1^, ^2^, ^3^ Both androgenic hormones and genetic predisposition have a role in AGA development, and the onset is therefore not expected in prepubertal patients without abnormal androgen levels.^3^, ^4^, ^5^ Over the last century, the onset of adrenarche and puberty have shifted toward younger ages, most probably due to hyperinsulinemic diet and increased environmental hormonal, and sexual exposure.^4^, ^5^ Thus, it is proposed that the prevalence of pediatric AGA is increased as this population is exposed to increased levels of circulating androgens at younger ages which leads to early development of AGA in genetically susceptible children.^2^, ^4^

Owing to its predilection for adults, AGA in the pediatric population is usually under-recognized, thus rarely discussed in the literature. Therefore, little is known about the natural history, clinical and trichoscopic characteristics, and associated diseases and hormonal abnormalities in pediatric patients with AGA.^1^, ^2^, ^3^, ^4^, ^5^, ^6^, ^7^ Free and total testosterone, dehydroepiandrosterone sulphate (DHEAS), and androstenedione are the most commonly evaluated androgens in those patients.^1^, ^2^, ^3^, ^5^ AGA, in itself, has been reported to be associated with many metabolic-related diseases such as metabolic syndrome (MetS), cardiovascular disease (CVD), insulin resistance (IR), hypertension, dyslipidemia, and obesity in adults.^8^, ^9^, ^10^, ^11^, ^12^, ^13^, ^14^, ^15^ Among them, MetS has received greater interest, and its prevalence has been found to be increased especially in patients with early-onset (age <35 years) AGA.^9^, ^10^, ^11^, ^12^, ^13^, ^15^ However, the presence of MetS or its risk factors in pediatric AGA has not been investigated before.

AGA is a life-long genetic process that may also affect the pediatric population. Therefore, it is critical to know its clinical presentation with the potential associated hormonal and metabolic abnormalities in this group of patients for timely diagnosis, appropriate medical intervention, and treatment. In this retrospective study, we evaluated the demographics, medical and family histories, clinical features along with the trichoscopic findings, androgenic hormones, and risk factors for MetS in pediatric patients with AGA.

## Methods

This study was approved by the Başkent University Institutional Review Board (Project no: KA 21/137), and all patients gave informed consent for the publication of their data.

The medical records of 23 pediatric patients diagnosed with AGA between December 2015 and June 2020 were reviewed retrospectively. The diagnosis of AGA was made by the characteristic patterned alopecia and the trichoscopic presence of >20% hair diameter diversity. The hair-pull test, trichoscopy, and laboratory examination (total blood cell count, blood levels of ferritin, and thyroid-stimulating hormone) were performed; the triggering factors (high fever, major surgical intervention, drugs, heavy diet, emotional stress) were questioned to exclude telogen effluvium and diffuse alopecia areata.

The patients’ ages and gender, medical and family history, age at disease onset, duration of AGA, and associated dermatological diseases were noted. The clinical and trichoscopic findings were recorded. The images were re-evaluated, and previously unrecorded features, if any, were added. The results of total blood cell count, blood levels of ferritin, thyroid-stimulating hormone, free and total testosterone, DHEAS, androstenedione, triglycerides, high-density lipoprotein (HDL) cholesterol, total cholesterol, low-density lipoprotein (LDL) cholesterol and fasting blood glucose, and homeostasis model assessment of insulin resistance (HOMA-IR) were recorded.

On their first admission, all the pediatric patients diagnosed with AGA were referred to pediatrics for the evaluation of sexual development and an underlying endocrine disorder. The blood pressure, body mass index (BMI), presence of early puberty, and signs of hirsutism or virilization were all recorded. The diagnosis of an endocrine or cardiovascular disorder such as diabetes mellitus, hyperandrogenism, polycystic ovary syndrome (PCOS), hyperlipidemia, hypertension, or MetS, if made, were also noted.

MetS risk factors were particularly noted for each patient as previously described.^16^, ^17^ These included IR (HOMA-IR >2.7), high fasting blood glucose (>100 mg/dL), overweight (BMI 25–30 kg/m^2^) or obesity (BMI ≥ 30kg/m^2^), high blood pressure (≥130/85 mmHg), and lipid abnormalities (triglycerides >150 mg/dL or HDL cholesterol <40 mg/dL).

Pediatric AGA was classified as childhood AGA and adolescent AGA if the onset occurred before puberty and postpubertal, respectively. For this purpose, we questioned the patients or their parents for the existence of breast development, pubic and axillary hair growth, menstruation, and ejaculation at the time of the beginning of hair loss. If we were unable to determine the pubertal status at the age of AGA onset, we defined an adolescent by age older than ten years (as proposed by World Health Organization).

## Results

The study included 12 females and 11 males with a mean age of 15.3 ± 2.1 years (range, 10–21 years). Sixteen patients had adolescent AGA (eight females and eight males; mean age: 16.3 ± 2.3 years; range, 11–21 years), and seven patients had childhood AGA (four females and three males; mean age: 12.9 ± 2.1 years; range 12–19 years). Table 1 displays age at presentation, age at onset, and duration of AGA.

**Table 1:** Age at presentation, age at onset, and duration of AGA in 16 patients with adolescent AGA, in seven patients with childhood AGA, and in a total of 23 patients with pediatric AGA.

Patients	Age at presentation, years (mean ± SD)	Age at onset, years (mean ± SD)	Duration, years (mean ± SD)
Pediatric AGA, n = 23	15.3 ± 2.1	12.1 ± 3.1	3.1 ± 2.2
Females, n = 12	15.8 ± 3.6	11.8 ± 3.6	4.0 ± 2.5
Males, n = 11	14.6 ± 2.1	12.5 ± 2.6	2.1 ± 1.2
Adolescent AGA, n = 16	16.3 ± 2.3	13.9 ± 1.6	2.4 ± 1.5
Females, n = 8	17 ± 3.1	13.9 ± 2	3.2 ± 1.7
Males, n = 8	15.6 ± 0.1	13.9 ± 1.1	1.6 ± 0.6
Childhood AGA, n = 7	12.9 ± 2.1	8.1 ± 1.5	4.7 ± 2.7
Females, n = 4	13.5 ± 1.9	7.8 ± 1.9	5.8 ± 3.1
Males, n = 3	12 ± 2	8.7 ± 0.6	3.3 ± 1.5

AGA, Androgenetic Alopecia; n, number.

A family history of patterned hair loss in a first-degree relative was present in nine (56.3%) of 16 patients with adolescent AGA (six males, three females). Six males had only their father, and two females had only their mother, and one female had both parents affected. One of the two males with no family history of AGA had associated acne, while the other had no clinical or laboratory abnormality. None of the patients with childhood AGA reported a family history in the first-degree relatives. Two females with adolescent AGA had a previous history of IR, and one female with adolescent AGA reported a previous history of PCOS. One of the females with IR also had associated hypertension.

Concurrent dermatological diseases were observed in 11 (47.8%) of 23 patients. Of those, eight patients (72.7%) (five males, three females) and three patients (27.3%) (two females, one male) had acne and seborrheic dermatitis, respectively. Hirsutism was noted in two females, one of whom also had seborrheic dermatitis. One of the males with acne and one of the females with seborrheic dermatitis, who were adolescents at initial presentation, had childhood AGA. The patients’ sexual development and growth parameters were appropriate for their ages except for the female with early puberty, hirsutism, and seborrheic dermatitis.

**Figure 1:**
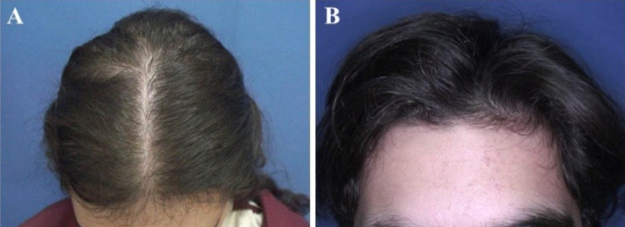
Hair loss patterns observed in pediatric androgenetic alopecia. (A), Diffuse thinning at the crown with preservation of the frontal hairline in a female with adolescent androgenetic alopecia. (B), Preservation of the frontal hairline in a male with adolescent androgenetic alopecia who has diffuse thinning at the crown.

**Figure 2:**
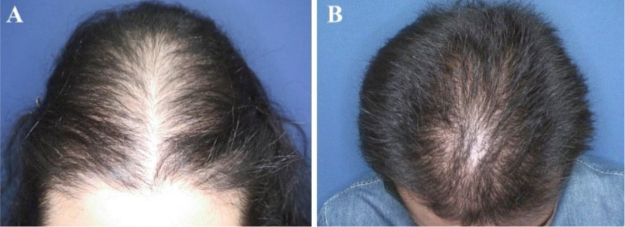
Hair loss patterns observed in pediatric androgenetic alopecia. (A), “Christmas tree” pattern in a female with adolescent androgenetic alopecia. (B), Bitemporal, frontoparietal and vertex thinning in a male with adolescent androgenetic alopecia.

The most common hair loss pattern was diffuse thinning at the crown with preservation of the frontal hairline (Figs. 1A and 1B) and was noted in 10 (43.5%) of 23 patients with pediatric AGA. It was followed by “Christmas tree” pattern (Fig. 2A) in four (17.4%), bitemporal, frontoparietal, and vertex thinning (Fig. 2B) in three (13%), diffuse thinning pronounced at the crown with bitemporal thinning in three (13%), bitemporal and vertex thinning in two (8.7%) patients, and diffuse thinning pronounced at the crown in one patient (4.4%). The hair pull test was negative in all patients. Six (54.5%) of 11 males had diffuse thinning at the crown with preservation of the frontal hairline. Among the females, the most frequent clinical presentations were diffuse thinning at the crown with preservation of the frontal hairline and “Christmas tree” pattern, each observed in four (33.3%) of 12 females (Table 2).

**Table 2:** Clinical and trichoscopic findings in 16 patients with adolescent AGA and in seven patients with childhood AGA.

Findings	Adolescent AGA, n (%)	Childhood AGA, n (%)
Clinical findings		
Diffuse thinning at the crown with preservation of the frontal hairline	6 (43.8) (3 M, 3 F)	4 (57.1) (3 M, 1 F)
“Christmas tree” pattern	3 (18.8) (3 F)	1 (14.3) (1 F)
Bitemporal, frontoparietal and vertex thinning	3 (18.8) (3 M)	0
Diffuse thinning pronounced at the crown with bitemporal thinning	1 (6.3) (1 F)	2 (28.6) (2 F)
Bitemporal and vertex thinning	2 (12.5) (2 M)	0
Diffuse thinning pronounced at the crown	1 (6.3) (1 F)	0
Trichoscopic findings		
>20% hair diameter diversity	16 (100)	7 (100)
Vellus hairs	14 (87.5)	7 (100)
Single-hair pilosebaceous units	6 (37.5)	2 (28.6)
Perifollicular discoloration	7 (4.8)	0
Yellow dots	6 (37.5)	1 (14.3)
Wavy hair	5 (31.3)	1 (14.3)
Circle hair	3 (18.8)	0
Dotted vessels	3 (18.8)	0
Honeycomb pigmentation	1 (6.3)	0
Thin arborizing vessels	1 (6.3)	0
Comma vessels	1 (6.3)	0

AGA, Androgenetic Alopecia; F, Female; M, Male; n, number.

**Figure 3:**
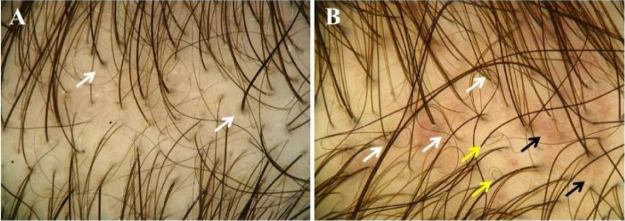
Trichoscopic findings of pediatric androgenetic alopecia. (A and B), Hair diameter diversity and single-hair pilosebaceous units. (A), Perifollicular discoloration (white arrows). (B), Wavy hair (white arrows), circle hair (yellow arrows), dotted and comma vessels (black arrows).

Trichoscopy (Figs. 3A and 3B) showed >20% hair diameter diversity in all 23 (100%) patients. Additional findings were vellus hairs (91.3%), single-hair pilosebaceous units (34.8%), perifollicular discoloration (30.4%), yellow dots (30.4%), wavy hair (26.1%), circle hair (13%), dotted vessels (13%), honeycomb pigmentation (4.4%), thin arborizing vessels (4.4%), and comma vessels (4.4%) (Table 2).

Fourteen patients (60.9%) with pediatric AGA (10 adolescent and four childhood AGA, eight females and six males) had at least one of the risk factors of MetS. Obesity or overweight was the sole MetS risk factor in eight patients (34.8%) (five adolescent and three childhood AGA, six females and five males). Of the remaining five patients with adolescent AGA, two were obese or overweight and had IR, one of whom also had hypertension, and two had high fasting blood glucose and IR, and one was obese and had high fasting blood glucose. One patient with childhood AGA had IR and lipid abnormalities (Table 3). Of the five patients with IR, three were females, and two were males. All three patients with high fasting blood glucose and one patient who had high blood pressure were females. The only patient with lipid abnormalities was a male.

**Table 3:** MetS risk factors in 16 patients with adolescent AGA, in seven patients with childhood AGA and in a total of 23 patients with pediatric AGA.

MetS risk factors	Adolescent AGA, n (%)	Chilhood AGA, n (%)	Total, n (%)
Overweight (BMI 25–30kg/m^2^) or obesity (BMI ≥ 30 kg/m^2^)	8 (50)	3 (42.9)	11 (47.8)
IR (HOMA-IR >2.7)	4 (25)	1 (14.3)	5 (21.7)
High fasting blood glucose (>100 mg/dL)	3 (18.8)	0	3 (13)
High blood pressure (≥130/85 mmHg)	1 (6.3)	0	1 (4.4)
Lipid abnormalities (triglycerides >150 mg/dL or HDL cholesterol <40 mg/dL)	0	1 (14.3)	1 (4.4)

AGA, Androgenetic Alopecia; BMI, Body Mass Index; HDL, High Density Lipoprotein; HOMA-IR, Homeostasis Model Assessment of Insulin Resistance; n, number; IR, Insulin Resistance; MetS, Metabolic Syndrome.

Other laboratory investigations showed increased total cholesterol levels in five patients (three childhood and two adolescent AGA), two of whom with childhood AGA also had increased LDL cholesterol levels. Of those five patients, three were obese or overweight. Blood levels of free and total testosterone and androstenedione were increased in two patients (one female and one male) and one female, respectively, all of whom had adolescent AGA. One patient with an increased blood level of free and total testosterone was overweight. The results of total blood cell count, blood levels of ferritin, thyroid-stimulating hormone, and DHEAS were within normal levels.

## Dıscussıon

Although pediatric AGA has rarely been reported in the literature, it is presumably not exceptional.^1^, ^3^, ^5^, ^6^, ^7^ In the pediatric population, AGA usually starts in adolescence but has also been reported to occur in prepubertal age.^1^, ^2^, ^3^, ^5^, ^6^, ^7^ Gonzalez et al.^1^ reported that, of the 438 pediatric patients complaining of hair loss, 57 patients were diagnosed with AGA, which was identified as the second most common diagnosis following alopecia areata in this population. Of those 57 patients, 52 patients were in adolescence, and AGA was the most prevalent type of alopecia in adolescents occurring in 42%. Additionally, a male predominance was noted overall and also in adolescent patients. Kim et al.^5^ observed 43 adolescents with AGA over a period of nearly five years, and males outnumbered females in their study, too. In both studies, age at onset and initial presentation were slightly higher in males rather than females.^1^, ^5^ In the series of Gonzalez et al.,^1^ males had a shorter delay to diagnosis, while Kim et al.^5^ reported a longer disease duration in males. Tosti et al.^3^ diagnosed 20 prepubertal children within four years. In their series, contrasting with the adolescent data, AGA was more frequent in females but started, and patients sought care at similar ages in both sexes. In their study, the youngest age at onset was six years, yet AGA was reported to begin in a patient as young as 5 years old.^3^, ^7^ Similar to previous data, in our series of pediatric patients, AGA was more common among adolescents. However, males and females were affected with similar frequency overall, also during adolescence and prepubertal ages. Differing from previous reports, age at initial presentation and AGA duration were higher in females, both in adolescent and childhood AGA. Age at onset was remarkably close to each other in males and females in adolescent AGA, while hair loss began at an older age in males with childhood AGA. Similar to previous reports, the earliest onset of AGA was 5 years of age in our study.

In men and some women, AGA results from dihydrotestosterone action, the 5-alpha reduced metabolite of testosterone, on a genetically susceptible hair follicle.^4^ Consequently, follicular miniaturization in the frontotemporal area and vertex in men and over the crown in women occurs, as these areas are more sensitive to the effects of androgens.^4^, ^13^ Clinically, most adolescent males with AGA exhibit hair thinning consistent with typical male pattern hair loss, as seen in adult men.^1^, ^2^, ^5^ However, a female pattern was recorded in 20% and 34% of males with adolescent AGA in two different studies.^1^, ^5^ Tosti et al. reported in their series of prepubertal children that in both sexes, AGA presented with a female pattern, consisting of thinning and widening of the central parting of the scalp with preservation of the frontal hairline.^2^ The observation of female pattern AGA in prepubertal children and adolescent males raised the belief that AGA may not be necessarily androgen-dependent, at least in some pediatric patients.^1^, ^3^, ^5^ In concordance with the previous studies, we also observed a female pattern in one-third of the males with adolescent AGA and all males with childhood AGA. However, interestingly, two females with childhood AGA and one female with adolescent AGA exhibited bitemporal thinning in addition to diffuse thinning pronounced at the crown. Our findings show that the clinical pattern of AGA in some of our pediatric patients was contrary to adult patterns, with males displaying a female pattern and females showing a male pattern. However, this observation has no precise explanation yet.

Trichoscopic findings in our pediatric patients with AGA were similar to those observed in adults.^18^ Besides >20% hair diameter diversity, which appears as a consequence of follicular miniaturization, increased proportion of vellus hairs, single-hair pilosebaceous units, perifollicular discoloration, yellow dots, and wavy hair were the most frequent findings. Trichoscopy also showed circle hair, honeycomb pigmentation, and most probably due to associated seborrheic dermatitis or as a normal scalp finding, dotted, comma, or thin arborizing vessels.

AGA is expected to begin after puberty when enough testosterone is available to be transformed into dihydrotestosterone.^1^, ^2^, ^3^, ^5^, ^6^, ^7^ As prepubertal children normally do not produce sufficient amounts of adrenal and gonadal androgens, the observation of AGA may indicate a hormonal abnormality in those patients.^1^, ^2^, ^3^, ^4^, ^5^ However, the study of Tosti et al.,^3^ a case report^7^ and likewise our study showed that AGA is not associated with increased blood levels of androgenic hormones beyond that expected for the stage of sexual development in prepubertal children. This observation suggests that AGA in children may not be gonadal androgen driven or that adrenal androgens may have a direct role.^1^, ^3^, ^7^ The adrenal secretion of androgen hormones begins to increase 2–3 years before puberty onset, called adrenarche, which occurs independently from gonadal maturation.^3^ It is proposed that the strong genetic predisposition, a characteristic common to the patients from the previous reports, could be responsible for an increased androgen sensitivity, and thus adrenal androgens may be adequate to induce AGA in predisposed children.^1^, ^3^, ^4^ The progressive lower age of onset of adrenarche could favor this process.^3^, ^4^ Yet, so far, the hypothesis of androgen hypersensitivity of the hair follicle in AGA has not been precisely proven in children and female pattern AGA. Therefore, either in children or adult women, AGA may have different pathogenetic pathways than the peculiarities of androgen metabolism underlying male pattern hair loss. In our study, none of our patients with childhood AGA had a positive family history in their first-degree relatives which also supports this suggestion.

AGA can sometimes be the sole dermatologic finding of PCOS.^2^ Although elevated androgen levels are found in a small subset of adolescent patients, PCOS is observed in nearly half of the adolescent females with AGA.^1^, ^4^ Strong family history is also noted frequently in both adolescent males and females with AGA.^1^, ^2^, ^5^ In the present study, in accordance with the literature data, AGA was reported in the first-degree relatives of more than half of our patients and nearly all males with adolescent AGA. Moreover, associated acne, seborrheic dermatitis, or hirsutism, which reflect the effects of androgens, were observed in nearly half of our pediatric patients, almost all of whom had adolescent AGA. However, hyperandrogenism was not a frequent finding overall, and PCOS or early puberty was noted only in two females. As proposed previously, we also think that, like in children with prepubertal testosterone levels, adrenal androgens might contribute to the development of AGA, or it might have occurred by a mechanism completely independent of the effect of androgenic hormones in our adolescent females with normal hormone profiles.

MetS describes clustering of central obesity, disturbed glucose metabolism, hypertension and dyslipidemia, and the presence of three or more of these components significantly increases the risk for developing CVD and diabetes.^8^, ^12^, ^17^ There are many studies that show the relationship between AGA and MetS and its individual components in adults.^8^, ^9^, ^10^, ^11^, ^12^, ^13^, ^14^, ^15^ In particular, early-onset of AGA in adults (before the age of 35 years) has been shown to be a risk factor for early onset of severe CVD, and some studies found a higher prevalence of IR and MetS in this group of patients.^8^, ^11^, ^13^, ^15^ The underlying mechanism linking AGA and MetS has not been fully established. However, it has been claimed that IR, the key mechanism in the development of MetS, could also contribute to AGA.^8^, ^13^, ^19^ Hyperinsulinemia may play a role in local androgen production from cholesterol and through local conversion of testosterone to dihydrotestosterone.^8^, ^19^ Vasoactive substances produced in IR further cause vasoconstriction and tissue hypoxia at the follicular level and contribute to the miniaturization of hair follicles.^8^, ^13^, ^19^ Moreover, high serum androgen levels and peripheral sensitivity to androgens observed in AGA may increase the risk of developing hypertension via androgen-mediated receptors found in the arterial wall endothelium and play an additional role in the association of AGA and MetS.^8^, ^13^

As in adulthood, obesity, and hence IR in childhood and adolescence, poses a growing problem, and pediatricians have increasingly diagnosed MetS in recent years.^20^, ^21^ Suffering from MetS and its risk factors during the pediatric age does not predict that they will persist in adulthood.^20^ Yet, the importance of early identification of children and adolescents at risk of developing MetS and subsequently progressing to diabetes and CVD in later life must not be underestimated.^20^, ^21^ Despite many studies in adults exist, the association of AGA and MetS in the pediatric population is yet to be investigated. In our study, more than half of the pediatric patients with AGA exhibited at least one MetS risk factor. The frequency of these risk factors was similar in females and males. Obesity or overweight, the most common cause of IR and the major determinant of MetS,^16^, ^17^, ^19^ was observed in nearly half of the patients and was the sole risk factor for one-third. IR and high fasting glucose which are the signs of disturbed glucose metabolism, were the second most commonly noted MetS risk factors. Other risk factors, including hypertension and lipid abnormalities, were infrequent in our pediatric patients. As our study did not have a control group, it is hard to determine whether a true association between pediatric AGA and the presence of MetS risk factors exists or not. However, we think that our findings are critical to underline the necessity for screening pediatric patients with AGA for the risk factors of MetS since most of these disorders are asymptomatic but related to later CVD and diabetes.

The retrospective nature and the lack of a control group were the main limitations of the current study. Additionally, the findings observed in adolescent and childhood AGA and also in females and males could not be statistically compared due to the insufficient number of patients.

## Conclusıon

The results of our study show that AGA is more prevalent among adolescents than children in the pediatric population and presents with clinical and trichoscopic features similar to those reported previously. Hyperandrogenism is not a common finding and probably does not contribute to the occurrence of AGA in the majority of children and adolescents. In contrast, genetic predisposition may play a role in adolescent AGA. Pediatric patients with AGA often present with at least one of the risk factors of MetS. Hence, we think that AGA in pediatric patients may be a sign of a future MetS, and therefore warrants an accurate and prompt diagnosis for early screening and halting the progression of this syndrome. Prospective controlled studies will better clarify the association of pediatric AGA with MetS risk factors.

## Financial support

None declared.

## Authors' contributions

Deren Özcan: Approval of the final version of the manuscript; critical literature review, data collection, analysis, and interpretation; effective participation in research orientation intellectual participation in propaedeutic and/or therapeutic management of studied cases; critical manuscript review; preparation and writing of the manuscript, study conception, and planning.

## Conflicts of interest

None declared.
